# Gestational Diabetes Independently Increases Birth Length and Augments the Effects of Maternal BMI on Birth Weight: A Retrospective Cohort Study

**DOI:** 10.3389/fped.2014.00112

**Published:** 2014-10-17

**Authors:** Magdalena Byström, Anthony Liu, Ann Elizabeth Quinton, Bernard Linton Champion, Kristy Mann, Michael Peek, Ralph Kay Heinrich Nanan

**Affiliations:** ^1^Sydney Medical School – Nepean, The University of Sydney, Penrith, NSW, Australia; ^2^Charles Perkins Centre – Nepean, The University of Sydney, Penrith, NSW, Australia

**Keywords:** birth weight, birth length, head circumference, gestational diabetes mellitus, maternal BMI

## Abstract

**Objective:** To investigate the effect of the interaction between gestational diabetes mellitus (GDM) and maternal body mass index (BMI) on the individual neonatal growth parameters.

**Design:** Retrospective cohort study.

**Setting:** A tertiary maternity service in Sydney, Australia, between 2005 and 2009.

**Population:** A cohort of 8859 women.

**Methods:** Generalized linear models.

**Main outcome measures:** Neonatal growth parameters, represented by *z*-scores for infant birth weight (BW), birth length (BL), and head circumference (HC) in GDM and non-GDM groups.

**Results:** Only GDM alone had an independent and positive effect on BL (*p* = 0.02) but not on BW or HC. In addition, in pregnancies complicated with GDM, the association between maternal weight and BW was significantly stronger (*p* < 0.001). In combination, GDM and maternal BMI significantly affected *z*-score differences between BW and BL (*p* < 0.001), in that underweight mothers had babies that were lighter relative to their length and inversely obese mothers had babies that were heavier relative to their length.

**Conclusion:** GDM independently influences BL and increases the association between maternal BMI and BW. In accordance with the hypothesis of the fetal origins of health and disease, the pronounced effects of GDM on fetal growth patterns demonstrated in this study are likely to influence long-term health outcomes in children.

## Introduction

Fetal growth is determined by a complex interaction between genetic factors and the *in utero* environment (1). Changes in the *in utero* metabolic environment can be caused by both gestational diabetes mellitus (GDM) and maternal weight.

Effects on the fetus including those that effect growth are not only associated with short-term complications for the neonate but are also associated with health outcomes in later life. For example, the Dutch famine study demonstrated a clear link between fetal growth and a range of diseases, including cardiovascular disorders (2). This concept has been coined “The Developmental Origins of Health and Disease (DOHaD)” and was previously summarized under the Barker Hypothesis (3, 4).

A well established and important *in utero* determinant of birth weight (BW) is GDM (5). In this condition, insulin resistance and failure of β-cell compensatory mechanisms causes increased levels of maternal glucose and lipids. The elevated substrates are transported across the placenta overexposing the developing fetus to nutrients, causing an increase in fetal growth (6). GDM affects approximately 5–10% of pregnancies in Western nations (7).

Separate to GDM, maternal obesity may also be characterized by insulin resistance and elevated lipids, allowing excess nutrients to be transferred across the placenta to the fetus contributing to fetal growth (1). Maternal obesity has been described to have similar effect on BW as GDM (8, 9). Approximately one-third of pregnancies are affected by maternal obesity, and in the female Australian population 2011–2012, approximately 56% were classified as overweight or obese (1, 10).

Apart from BW, fetal growth can also be determined by birth length (BL) and head circumference (HC). To be able to describe growth in more detail, it is also important to acknowledge the relationship of these parameters to each other as a measure of fetal growth.

Therefore, the aim of this study was to investigate the effect of the interaction between GDM and maternal body mass index (BMI) on individual neonatal growth parameters and their relationship to each other.

## Materials and Methods

**Figure 1:**
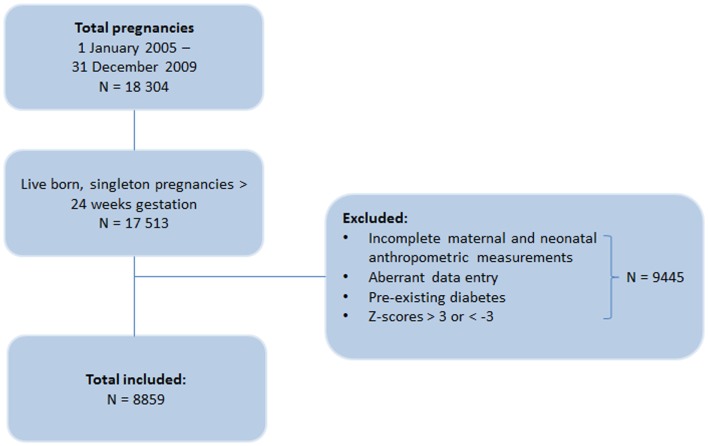
**Flow chart of the inclusion of pregnancies in the study**.

This is a retrospective cohort study of deliveries from a tertiary maternity service in Metropolitan Sydney, New South Wales, Australia, occurring between 2005 and 2009. Women were identified using the hospital’s obstetric database. The database is updated and maintained during and after pregnancy, and contributes to statewide data collection. The database identified 18,304 pregnancies, of which all live-born and singleton pregnancies with gestational age greater than 24 weeks were eligible for inclusion in the study (Figure 1). However, cases were excluded if the data were incomplete for maternal and neonatal anthropometric measurements or contained data entry errors (*n* = 9235). Mothers with pre-existing diabetes were further excluded from the study (*n* = 41).

Maternal characteristics extracted from the database included weight and height measured at the booking visit, age, parity, smoking status, and obstetric complications including GDM and pre-eclampsia. Maternal BMI, calculated as kg/m^2^, were divided into six BMI ranges according to the WHO-criteria: underweight (<18.5 kg/m^2^), normal weight (18.5–24.9 kg/m^2^), overweight (25–29.9 kg/m^2^), obese class I (30–34.9 kg/m^2^), obese class II (35–39.9 kg/m^2^), and obese class III (≥40 kg/m^2^) (11). Maternal smoking was self-reported and included smoking at any time during pregnancy.

All pregnant women attending the antenatal clinic were offered screening for GDM, which was done at 24–28 weeks of gestation. Depending on the medical or family history, a 50 g glucose challenge test (GCT) or a 75 g 2 h oral glucose tolerance test (OGTT) was conducted. Women were considered high risk for GDM if the 1-h GCT was >7.8 mmol/L with the diagnosis being confirmed by subsequent 2 h OGTT. GDM was diagnosed based upon contemporaneous Australasian Diabetes in Pregnancy Society (ADIPS) criteria; fasting glucose during OGTT ≥ 5.5 mmol/L or 2-h glucose was ≥8.0 mmol/L (12).

Neonatal variables extracted from the database included gestational age at birth, BW, BL, and HC. Gestational age, in weeks, was estimated from the time of the last menstrual period (LMP), and was confirmed by ultrasonography. If there was a greater than 10-day difference between the estimated age by LMP and the estimated age on second trimester ultrasonography, the age calculated by ultrasonography was used.

*z*-scores, adjusted for gestational age, were calculated for BW, BL, and HC without customizing for maternal characteristics (13).

### Studied outcomes

The outcomes studied were neonatal growth variables, represented by gestational age adjusted *z*-scores for infant BW, BL, and HC. To exclude biologically unrealistic values, the outcome variables were further restricted; infants were excluded from the analyses if any of the *z*-scores were >3 or <−3 standard deviations from the expected mean (*n* = 169). This was applied to avoid outliers in documentation or potential other pathologies either not or inaccurately listed in the database.

To obtain the neonatal growth proportionality, the difference between the different *z*-scores was calculated: 1: HC minus BW (HC − BW); 2: BW minus BL (BW − BL); and 3: HC minus BL (HC − BL). For example, a baby might have a BW *z*-score of +1 (+1 SD from its expected mean), but a HC of −1 (−1 SD from its expected mean), which results in a difference of 2 between the two outcome variables, which would be a surrogate for an asymmetric growth pattern. A perfect symmetric growth pattern would result in a difference of 0.

### Statistics

Descriptive statistics are reported as mean ± SD and median and interquartile range. Maternal characteristics and pregnancy outcomes were compared between GDM and non-GDM women using a Students *t*-test for normally distributed continuous data, a Mann-Whitney *U*-test for skewed continuous data or a chi-squared test for categorical data.

Generalized linear models were used to examine the effect of GDM (yes/no) on the studied outcomes. The models were adjusted for maternal age, maternal BMI (categorical), parity, smoking (yes/no), maternal pre-eclampsia and hypertension, gestational age, and infant gender. Additionally, an interaction term between GDM and maternal BMI was investigated in each of the outcome models. The adjusted means in each GDM group from these models were plotted over the maternal BMI categories.

SAS Version 9.3 software (SAS Institute Inc., Cary, NC, USA) was used for all statistical analyses. A *p*-value of <0.05 was considered statistically significant. No adjustment was made for multiple comparisons.

### Ethics

The study was approved by the Local Human Research Ethics Committee of the Nepean Blue Mountains Local Health District on 18 March 2013 (HREC no. 10/16).

## Results

### Demographics

The study included 8859 women, of whom 498 (5.6%) patients had GDM. Maternal characteristics and pregnancy outcomes for GDM and non-GDM women are shown in Table 1.

**Table 1:** **Maternal characteristics and pregnancy outcomes in GDM and non-GDM women**.

Characteristics	GDM (*n* = 498)	Non-GDM (*n* = 8361)	*p* value
Maternal age (years)	31.6 ± 5.2	28.6 ± 5.3	<0.001
Maternal BMI (kg/m^2^)	32.0 [24.3–35.0]	24.8 [21.5–29.7]	<0.001
Parity	1.0 [1.0–2.0]	1.0 [1.0–2.0]	0.034
Smoking (%)	99 (19.9)	2208 (26.4)	0.002
Hypertension (%)	40 (8)	418 (5)	0.004
Pre-eclampsia (%)	13 (2.6)	163 (2)	0.4
Gestational age (weeks)	39.0 [38.2–39.5]	39.5 [38.6–40.4]	<0.001
Birth weight (g)	3384 ± 532	3455 ± 521	0.003
Birth length (cm)	51.0 [49.0–53.0]	51.0 [49.5–53.0]	0.5
Head circumference (cm)	34.5 [33.5–35.5]	34.5 [33.5–35.5]	0.9
BW *z*-score	0.29 ± 1.1	0.09 ± 1.0	<0.001
BL *z*-score	0.18 ± 1.0	−0.02 ± 0.94	<0.001
HC *z*-score	0.12 ± 0.89	−0.04 ± 0.86	<0.001

*Values are shown as mean ± SD, median [Q1–Q3] or *n*(%)*.

*cm, centimeter*.

In pregnancies complicated with GDM, mothers were older and had higher average BMI. They were also less likely to smoke. Examining the *z*-scores, adjusted for gestational age, there were significant differences for BW, BL, and HC between the two groups, where GDM women gave birth to babies that were heavier, longer, and had greater HC compared to babies born to non-GDM women. Table 2 summarizes birth parameters for all BMI groups.

**Table 2:** **Birth parameters according to BMI categories**.

		BMI categories^a^						*p* value
		1	2	3	4	5	6	
Total	BW *z*-score	−0.36	−0.03	0.18	0.27	0.41	0.50	<0.001
	BL *z*-score	−0.24	−0.08	0.03	0.10	0.17	0.20	<0.001
	HC *z*-score	−0.34	−0.15	0.00	0.12	0.26	0.39	<0.001
GDM	BW *z*-score	−0.83	−0.12	0.26	0.46	0.55	1.03	<0.001
	BL *z*-score	−0.05	−0.09	0.26	0.20	0.29	0.54	<0.01
	HC *z*-score	−0.46	−0.14	0.04	0.19	0.33	0.69	<0.001
No GDM	BW *z*-score	−0.35	−0.03	0.18	0.25	0.39	0.43	<0.001
	BL *z*-score	−0.25	−0.08	0.02	0.09	0.15	0.16	<0.001
	HC *z*-score	−0.34	−0.15	0.00	0.12	0.25	0.35	<0.001

**z*-scores reported in means*.

*BW, birth weight; BL, birth length; HC, head circumference*.

*^a^BMI category 1: <18.5 kg/m^2^; 2: 18.5–24.9 kg/m^2^; 3: 25–29.9 kg/m^2^; 4: 30–34.9 kg/m^2^; 5: 35–39.9 kg/m^2^; 6: ≥40 kg/m^2^*.

### The independent effect of gestational diabetes on growth

Examining the independent effect of GDM on the neonatal growth variables, i.e., *z*-scores for BW, BL, and HC, models adjusted for maternal age, maternal BMI, parity, smoking status, hypertension, pre-eclampsia, gestational age, and infant gender, were used. The results show that GDM only had an independent effect on BL *z*-score (*p* = 0.02). Excluding GDM, there is an independent effect of BMI on *z*-scores for BW, BL, and HC (*p* < 0.001).

### The interaction effect between GDM and maternal BMI

**Figure 2:**
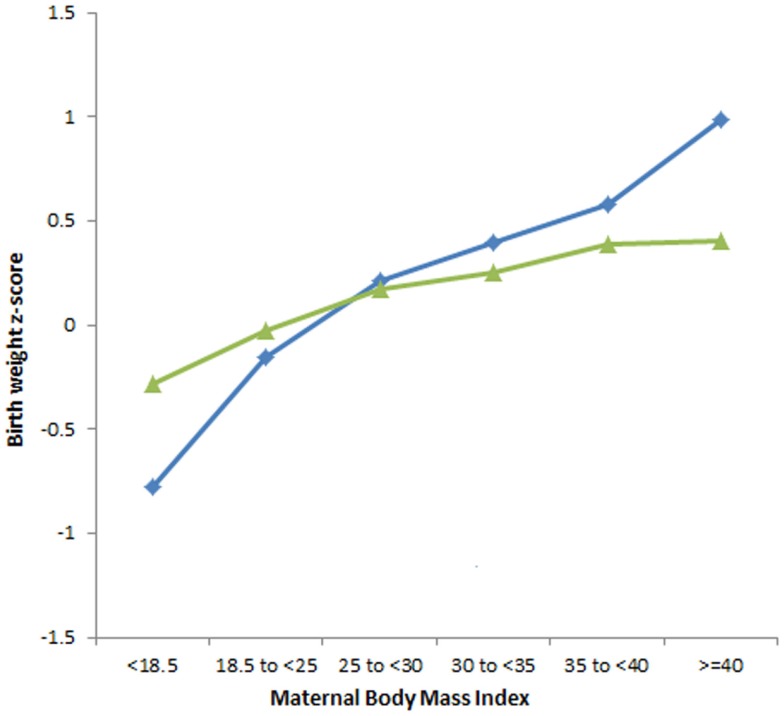
**The association between increasing maternal BMI and BW *z*-score in babies of GDM and non-GDM women, adjusted for all confounding factors**. Interaction GDM × maternal BMI (*p* < 0.001). **GDM** 
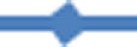
,**non-GDM** 
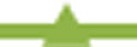
.

The interaction effect between GDM and maternal BMI on the neonatal growth variables was examined. Figure 2 compares the association between increasing maternal BMI and BW *z*-score in women with and without GDM after adjusting for all confounding factors. The increase in BW with increasing maternal BMI is more pronounced in pregnancies with GDM than in non-GDM pregnancies (*p* < 0.001).

There was no interaction effect between maternal BMI and GDM on BL *z*-scores (*p* = 0.19) and HC *z*-scores (*p* = 0.27).

### Effect of GDM on intra-individual length and weight *z*-scores

**Figure 3:**
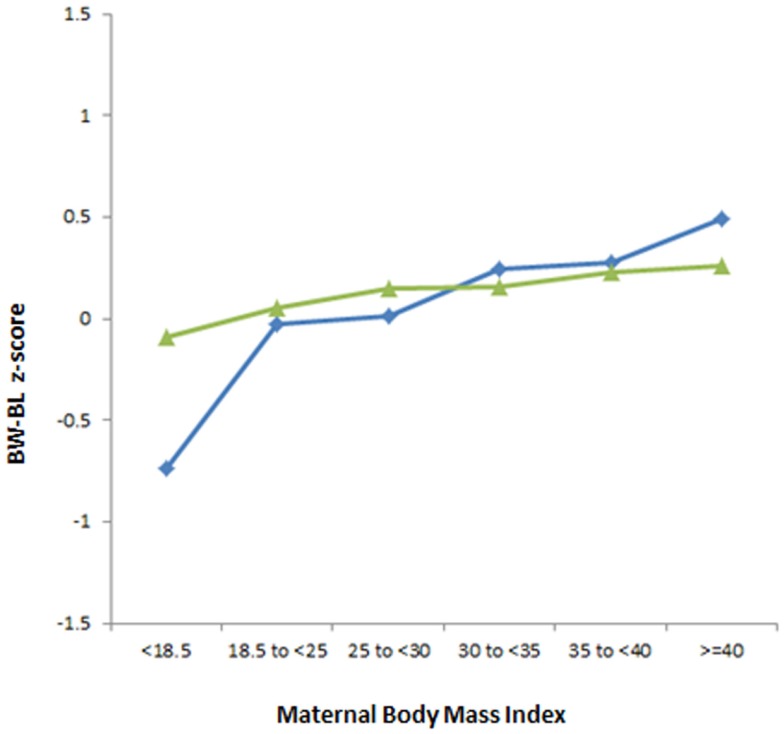
**The association between increasing maternal BMI and the mean differences between BW and BL *z*-scores in GDM and non-GDM women**. Interaction GDM × maternal BMI (*p* < 0.01). **GDM** 
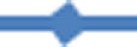
,**non-GDM** 
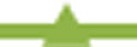
.

As we have described an independent effect of GDM on length *z*-scores and a combined effect of GDM and maternal BMI on weight *z*-score, we examined the effect of GDM on the relationship between weight and length *z*-scores (Figure 3). In GDM women with BMI < 18.5, the growth asymmetry is enhanced with babies having a mean difference of almost 0.74 between their BW and BL *z*-scores. The interaction effect of maternal BMI and GDM on the relationship between BW and BL *z*-scores (BW-BL) is highly significant (*p* < 0.001).

## Discussion

### Main findings

This retrospective study has confirmed that the association between maternal weight and BW is more pronounced in pregnancies complicated with GDM. We have also found that GDM together with maternal BMI affects the neonatal growth patterns, specifically the relationship between BW and BL. Lean GDM women gave birth to babies with less weight compared to their length. Conversely, obese GDM women gave birth to babies with greater weight compared to their length. Since the interaction between maternal BMI and GDM did not have an effect on the HC *z*-score or the BL *z*-score, it is proposed that the variation in BW, in relation to maternal BMI, contributes to the asymmetry among babies born to GDM women. In summary, the interaction between maternal weight and GDM does not seem to affect skeletal growth, only fetal weight.

### Strengths and limitations

The strength of our study is that this study has systematically compared the symmetry between BW, BL, and HC, between controls and pregnancies affected by GDM in relation to increasing maternal BMI.

Limitations to this study are its retrospective nature and the unavailability of measures of maternal blood glucose values, which have been shown to correlate to fetal and/or neonatal growth patterns (14). In addition, the effect of treatment modality on different growth parameters and growth proportion would be interesting to investigate. Furthermore, a potential confounding factor is that women with GDM were usually delivered electively at 38 weeks, and hence the natural progression of fetal proportions in these women beyond this gestational age could not be ascertained. Another area to consider is that we used booking weight (BMI) in our study. Since weight gain during pregnancy has been found to affect BW, it is possible that weight gain/loss in pregnancy might have affected our results (15). Unfortunately, our data collection system does not capture these parameters.

### Interpretation

The effect of GDM on BW has been demonstrated in previous studies (7, 16). In addition, the HAPO study found a strong and continuous correlation between maternal glucose levels and increased BW (5). Although our results showed that GDM did not have a significant independent effect on BW, we found that GDM did enhance the effect of maternal BMI on BW confirming previous observations (17, 18). Makgoba et al. investigated the interaction between GDM and maternal BMI on BW in white, black, and South Asian women. They found that the enhancing effect of GDM on the association between maternal BMI and BW differed between the racial groups, suggesting that part of the interaction effect between GDM and maternal BMI can be attributed to genetic factors (17). The vast majority of the cohort in this study was of Caucasian origin.

This interaction effect cannot completely be explained by genetics. Women who develop GDM show resistance to the action of insulin to stimulate glucose disposal and to suppress production of glucose and fatty acids, resulting in increased maternal plasma glucose levels (7). The exposure to increased maternal glucose supply stimulates fetal pancreatic insulin production, leading to accelerated fetal growth (19). However, circulating levels of other nutrients, such as triglycerides (TG) and free fatty acids (FFA), are also increased in GDM and may also contribute to fetal growth (1, 6, 20). In GDM, other alterations affecting fetal growth have been found, including increased placental weights, which could increase placental nutrient transfer, thereby increasing fetal nutrient supply (21). Other alterations include altered levels of adipokines correlated to BW (20, 22).

Maternal obesity, in the absence of GDM, has been found to have a strong independent relationship with BW and the pathophysiology may have similarities with GDM (8). Adipose tissue was originally considered to be a storage site for TG. However, adipose tissue is metabolically active with multiple endocrine and immune functions, with the potential to produce disturbances in insulin signaling pathways resulting in increased insulin resistance with increasing weight (19). Maternal obesity is also characterized by dyslipidaemia, which, together with insulin resistance and failure of β-cell compensatory mechanisms, leads to increased metabolic fuels allowing excess nutrients to be transferred across the placenta to the fetus (1). Larger placentas are also more likely to occur in obese women, with altered levels of adipokines, which may also be associated with fetal growth (1, 23).

Gestational diabetes and obesity have similar metabolic and structural alterations and may affect fetal growth in similar ways. When combining maternal weight and GDM, we found that the two variables together have an even more pronounced effect on BW than either variable alone.

When examining the interaction effect between GDM and maternal weight, we found that lean women affected with GDM gave birth to babies with less weight in relation to both the HC and BL compared to controls. We hypothesize that the insulin treatment given to lean women with GDM may have a greater effect on growth potential, and in comparison to obese women treated with insulin, lean women might not have been able to compensate for low blood glucose values with higher lipid values, which have also been found to affect fetal growth (20). In this context, treatment with insulin would be interesting to analyze in future studies.

Previous studies have investigated the independent effect of GDM on other growth parameters, such as HC and BL. However, these studies have either examined growth parameters during pregnancy only or analyzed the odds ratio of LGA in length among babies born to women with GDM (16, 24).

We found that the interaction between GDM and maternal BMI did not have a positive effect on BL or HC. Neither did GDM alone affect the HC. Nevertheless, GDM had an independent effect on BL.

## Conclusion

Gestational diabetes increases the association between maternal weight and neonatal BW. In addition, GDM together with maternal BMI affects the neonatal growth patterns more than either variable alone. We hypothesize the variation in BW, in relation to maternal BMI, contributes to the asymmetry among babies born to GDM women. How GDM in combination with maternal BMI and their effect on fetal growth patterns affect the well-being of the child later in life will need to be addressed in future studies.

## Author Contributions

All authors were involved in formulating the hypothesis and interpreting the data. All authors were involved in writing, editing, and approving the final manuscript. All authors agree to be accountable for all aspects of the manuscript.

## Conflict of Interest Statement

The authors declare that the research was conducted in the absence of any commercial or financial relationships that could be construed as a potential conflict of interest.
